# Is Gamification the New Panacea for Health Behavioral Changes? Implications for the Health and Life Insurance Industry

**DOI:** 10.2196/80684

**Published:** 2026-04-13

**Authors:** Abbas Salami, Tasos Papastylianou, Marvellous Adeoye, John Ronayne, Honor Bixby, Robert S Stawski, Bernard Fromson, Matt Doltis, Osama Mahmoud, Mariachiara Di Cesare

**Affiliations:** 1 Institute of Public Health and Wellbeing, University of Essex, Wivenhoe Park, Colchester, CO4 3SQ, United Kingdom; 2 School of Computer Science and Electronic Engineering, University of Essex, Wivenhoe Park, Colchester, CO4 3SQ, United Kingdom; 3 YuLife, London, United Kingdom; 4 School of Health and Medical Sciences, City St George's, University of London, London, United Kingdom; 5 Emma Eccles Jones College of Education and Human Services, Utah State University, Utah, UT, United States; 6 School of Mathematics, Statistics and Actuarial Science, University of Essex, Colchester, United Kingdom; 7 Department of Applied Statistics, Helwan University, Cairo, Egypt

**Keywords:** gamification, behavior change interventions, physical activity, health and life insurance, mobile phone, digital health

## Abstract

Chronic health conditions impose substantial financial and operational burdens on the public health sector and insurance providers in the United Kingdom. While gamification demonstrates the potential for enhancing health behavior, a structured analysis linking to established behavioral frameworks is missing. We provide a viewpoint on whether, as health and life insurers transition from traditional risk assessment toward proactive risk reduction strategies, gamification offers an innovative mechanism to strengthen their prevention initiatives and insurer-insured relationships. We examine how gamification aligns with key theoretical models, including the Behavior Change Wheel and Behavior Change Techniques, and how gamification elements can be mapped onto them. This enables combining multiple Behavior Change Techniques into effective interventions, which provide engaging user experiences and promote intrinsic motivation. We distinguish gamification from mere incentivization, highlighting its potential for sustained health outcomes. We also explore the ethical and practical considerations of gamification in the insurance sector. We highlight the need for a robust ethical framework that preserves an individual’s ability to make free and informed decisions, while ensuring inclusivity and absence of discrimination based on personal characteristics that may affect their capacity to engage in healthy behaviors. Similarly, we highlight how privacy, transparency, and accountability need to be prioritized in the governance structure of gamification programs in the sector. Our analysis emphasizes that gamification has the potential to represent the new panacea for the insurance sector, if effective gamified interventions incorporate inclusive design principles, theoretical grounding, ethical accountability, and continuous refinement to ensure alignment with long-term public and individual health objectives. This viewpoint is the first to map gamification and behavioral change frameworks into a unified model for insurer-led health behavior interventions and encourage greater investment in gamified wellness products and the use of theory-driven behavioral science in insurance-led digital health tools.

## Introduction

In the United Kingdom, the increasing prevalence of chronic health conditions is placing a growing financial and systemic burden on the public health sector and insurance providers. An estimated 15 million people in the country live with long-term conditions such as heart disease, cancer, renal disease, and diabetes, which cost the National Health Service (NHS) £73.3 billion in 2023 alone [1,2] (for comparison, at the time of writing, the conversion rate was approximately GBP £1=US $1.32). A significant factor contributing to this issue is physical inactivity [3]. In the United Kingdom, 32% of men and 40% of women aged above 18 years did not meet physical activity (PA) recommendations in 2022; for adults aged above 70 years, this rose to 47% and 56%, respectively, and for adolescents aged 11 to 17 years, this rose to a staggering 70% and 85%, respectively [4], despite strong evidence that regular PA reduces the risk of mortality and morbidity [5]. Even in otherwise phenotypically healthy individuals, sedentary lifestyles are more likely to be associated with early physiological changes indicating metabolic stress, which may predate overt metabolic disease [6,7]. In 2022, physical inactivity cost the NHS an estimated £0.9 billion [8]. Beyond direct medical costs, the overall economic burden, including diminished productivity and quality of life, was estimated at £7.4 billion per year [8]. Globally, the annual cost of preventable chronic conditions associated with physical inactivity amounted to approximately US $27 billion [4]. These trends intensify pressures on the NHS and contribute to rising insurance claims and costs, especially within an aging and chronically ill population.

Health behaviors are actions taken by individuals that affect their health and encompass a wide range of actions such as PA, smoking, or diet [9]. Therefore, behavior change is a critical component of public health initiatives aimed at improving individual and community well-being. However, multiple barriers, such as lack of motivation, limited knowledge, and insufficient support, can prevent individuals—particularly middle-aged adults—from engaging in and maintaining healthy behaviors [10].

Traditional approaches to health behaviors often rely on education and awareness campaigns. While valuable, these often fail to achieve sustained behavior change [11], as individuals often struggle to adapt their daily routines accordingly [12]. This knowledge-action gap highlights the need for innovative interventions that can effectively engage individuals and facilitate lasting change.

In this context, gamification, defined as “the use of game design elements in nongame contexts” [13], has emerged as a novel and promising approach [14]. It leverages the intrinsic enjoyment associated with games to engage users, making health-related tasks more appealing and rewarding [15]. By incorporating elements such as points, badges, leaderboards, and challenges, gamification can transform healthy activities into engaging experiences that encourage sustained participation [16]. Leveraging social interaction is a key component of gamified interventions, fostering a sense of community and competition through elements such as leaderboards, team challenges, and shared goals [15,17]. These features engage individuals’ intrinsic motivations for social connection, recognition, competition, and belonging [18,19]. Similarly, collaborative tasks or group challenges encourage participants to coordinate their efforts with others, transforming individual behaviors into socially reinforced habits [20,21].

While gamification is often paired with incentivization [22], these reflect fundamentally different strategies. Incentivization encourages behavior change primarily through extrinsic motivators, such as financial rewards [23,24]. Gamification focuses more on creating engaging, immersive experiences beyond mere incentivization by additionally using intrinsic motivators such as enjoyment, mastery, and autonomy [25]. For instance, while incentivization might reward improved fitness with monetary discounts, gamification could further motivate through level unlocking and badge earning. Similarly, gamification transcends mere incentivization by embedding social mechanisms and engaging deeper motivational drivers related to personal identity, mutual support, and social recognition [26]. Therefore, although both approaches can be effective [22], gamification uniquely fosters sustained engagement by making the behavior change process inherently enjoyable rather than simply driven by external reward prospects [27].

The rapid advancement of computing technology has revolutionized the landscape of health behavior change, resulting in a diverse array of consumer applications aimed at monitoring and maintaining individual health [28,29]. This has given rise to the cultural phenomenon known as the “quantified self” [30-32], whereby individuals use technology, such as activity trackers or sleep monitors, to track and quantify various aspects of their daily lives. These technological advancements have provided policymakers with new avenues to implement effective behavioral interventions, catering to both individual- and population-level health initiatives [33]. Digital health technologies have provided a fertile ground for integrating gamification into behavior interventions, offering scalable solutions with real-time monitoring, feedback, and personalization to sustain user engagement [34,35]. The global gamification market is projected to grow from US $22.01 billion in 2024 to US $73.66 billion by 2029, with the mobile segment leading the market [14,36]. In addition, gamification has attracted interest in sectors such as health and life insurance (Textbox S1 in Multimedia Appendix 1 [22,37-90]), where it holds the potential to drive positive health behaviors among policyholders while reducing risks and costs for insurers.

This paper aims to explore the role of gamification in supporting health behavior change, with a particular focus on its emerging use in the health and life insurance industry and the implications for the sector. To support this discussion and inform the design of gamified products in insurance settings, we outline the theoretical foundations of gamification and review examples of its application in health behavior change interventions (BCIs), particularly in gamified digital health applications. We also discuss potential benefits and challenges, as well as ethical and practical considerations involved in designing such interventions within this setting. While the primary audience of this work is the health insurance sector, the discussion is also relevant to other stakeholders involved in designing health behavior–oriented interventions more generally.

It should be noted that, more broadly, the scope of health apps extends beyond behavior-oriented applications; apps addressing treatment, education, and advice are also common [91-93] and have implications for the health and life insurance industry. However, these are generally unrelated to gamification and, therefore, beyond the scope of this paper.

## The Evolving Role of Health and Life Insurers: From Risk Prediction to Risk Reduction Through Health Behavior Change

Traditionally, health insurance underwriting involves point-in-time evaluations using demographic, medical, behavioral, and sometimes financial data to assign individuals to risk classes, which then determine premiums [94,95]. For employer-sponsored group cover, traditional underwriting relies on broad rating factors, such as age, occupation, and group-level claims experience. This approach spreads risk across a company’s workforce, enabling employees below specified benefit thresholds to receive cover without individual medical underwriting [96]. While this model is administratively efficient and equitable at scale, the data collected during underwriting remain static until policy renewal. In addition, insurers typically refine their pricing models only periodically, drawing on population-level experience. However, in recent years, there has been a shift toward more dynamic approaches enabled by technology and artificial intelligence [97]. Insurers may leverage data from smartphone or wearable health apps to monitor risk-related behavioral and biometric data in real time [98]. Rather than simply predict claims risk, the goal is to reduce risk by promoting healthier behaviors, shifting insurers from being passive underwriters to partners in health management.

Several health insurers in the United States (eg, UnitedHealth, Humana, Cigna, and Aetna) and the United Kingdom (eg, Vitality, YuLife, Aviva, and AXA Health) now offer mobile apps and wellness programs (refer to Textbox S1 in Multimedia Appendix 1 for specific examples) to encourage healthier lifestyles among policyholders. These initiatives aim to create a healthier policyholder pool and support broader organizational outcomes, such as reduced absenteeism and improved productivity. This is particularly relevant as emerging risks such as mental health represent a sizable share of group income protection claims in the United Kingdom, accounting for 21% of new claims in 2023 [99].

These insurer-led programs are often primarily reward based, relying on financial incentives or discounts to motivate engagement. As rewards also constitute a core element of gamification, it is important to clarify how such mechanisms operate within gamified health interventions, as opposed to nongamified approaches that rely solely on monetary incentives. Addressing this distinction requires an understanding of how intrinsic and extrinsic motivations interact in this context. Extrinsic motivation, including points or prizes granted upon completing specific tasks, forms one facet of gamification. In contrast, intrinsic motivation is driven by the pleasure and satisfaction derived from immersive, enjoyable game participation, such as eagerness to compete [37]. Although external rewards can catalyze short-term motivation spurts, overreliance on them can diminish intrinsic motivation [100]. Furthermore, depending solely on extrinsic motivators may hinder sustainable engagement or result in negative consequences, such as cheating or disengagement [101].

Therefore, understanding when and how gamification supports sustainable behavior change requires grounding these design elements within established theories and frameworks of behavior change.

## Theoretical Foundations of Health Behavior Change and the Role of Gamification

Changing health behaviors presents a significant challenge across populations [102]. To address its complexity, several theories, collectively referred to as *theories of behavioral change,* have been developed to provide deeper insights into the processes underlying changes in health behavior and facilitate the design and evaluation of BCIs [102]. These theories vary in scope, from models focusing on cognitive risk appraisal to those emphasizing motivation, capability, and environmental influences (Textbox S2 in Multimedia Appendix 1).

### From Theory to Intervention Design

#### Overview

While these theories provide valuable insights into why people engage or fail to engage in health behaviors, highlighting the roles of beliefs, perceived capability, motivational quality, and situational triggers, they offer limited guidance on how to systematically design interventions that can modify these determinants in practice [103]. To bridge this gap, comprehensive frameworks such as the Behavior Change Wheel (BCW) and the Behavior Change Technique (BCT) taxonomy offer systematic guidance on translating theory into practice.

#### The BCW Framework

The BCW framework [87] has been developed to design interventions by linking them to a detailed analysis of the behaviors they aim to influence. Consisting of 19 distinct behavior change frameworks, the BCW effectively captures mechanisms (internal and external) potentially involved in changing behaviors. For example, internal factors may involve motivation or physical ability, while external factors can include social norms or access to resources. Three essential components—capability, opportunity, and motivation—at the center of the BCW framework (known as the capability, opportunity, motivation, and behavior [COM-B] system) are needed for change to occur (Textbox S3 in Multimedia Appendix 1). At the outer level, the BCW framework outlines 9 distinct categories of BCIs, each targeting 1 or more components to drive behavior change [104] (Table S1 in Multimedia Appendix 1). The BCW has emerged as a widely accepted framework for designing effective health behavior interventions across various settings. The UK public health guidance encourages local authorities to adopt this framework to systematically develop population-level interventions [105]. Similarly, international organizations such as the United Nations Children’s Fund emphasize the importance of the BCW in ensuring evidence-based and context-sensitive programming in global health initiatives [106]. For example, the BCW has been used in adults with obesity to create tailored home-based exercise programs [107]; in the workplace to address sedentary behavior by identifying key influences on employee behavior and linking them to appropriate intervention strategies [108]; in maternal health to guide the co-design of the Healthy Gut Diet initiative, which aims to prevent gestational diabetes through changes in diet [109]; and to develop resources for enhancing PA conversations in health care settings [110].

#### The BCT Taxonomy

Health behavior interventions tend to use multiple interconnected components [111]. Consequently, more nuanced theoretical constructs have been developed to inform the design of effective behavior change strategies. Notably, the BCT taxonomy version 1 [90] identifies 93 distinct “active ingredients” or techniques that can potentially be integrated into an intervention. These techniques are observable, replicable, and irreducible components designed to modify or redirect the causal processes that govern behavior [112,113]. The taxonomy serves as a comprehensive, hierarchical classification system of intervention components using 93 BCTs, categorized into 16 clusters (Table S2 in Multimedia Appendix 1). For instance, the concept of incentives is classified as a BCT under the “reward and threat” cluster. This taxonomy effectively codes interventions across various behavioral domains [114], such as PA and dietary habits [115,116].

Several of the BCW-based interventions discussed earlier provide clear examples of the BCT approach. For instance, Power et al [107] integrated 24 BCTs into their home-based exercise program for adults with overweight and obesity, including *goal setting*, *action planning*, and *reward* systems to promote sustained engagement. Ojo et al [108] identified 39 BCTs to reduce workplace sitting, targeting both motivational and contextual barriers through techniques such as *environmental restructuring*, *prompts and cues*, and *social support*. Meloncelli et al [109] incorporated 40 BCTs into the Healthy Gut Diet intervention from several BCT clusters, including *goals and planning*, *feedback and monitoring*, and *reward and threat,* to encourage healthier eating during pregnancy. These examples demonstrate how BCW-guided interventions can also systematically embed multiple BCTs to address complex health behaviors effectively.

## Linking Gamification to Theories of Behavioral Change

Earlier, we introduced the 9 intervention functions of the BCW framework and discussed how they address deficits in the components of the COM-B system. We also argued that the techniques outlined in the BCT taxonomy are linked to the BCW’s intervention functions. In essence, intervention functions can consist of 1 or more BCTs, and a single BCT can serve multiple intervention functions [87]. As game elements can be straightforwardly mapped onto corresponding BCTs, gamification can serve as a driver for effective behavioral change.

For instance, points and badges serve as *nonspecific incentives* and *rewards*, respectively, offering a sense of achievement, while points can also become *material incentives* if redeemable [117]. Leaderboards encourage *social comparison* and *competition* [19], motivating users by letting them track their progress against others. Levels and challenges reflect BCTs related to *goal setting* and *graded tasks*. Each level typically sets a specific, achievable goal (reaching a daily step count or avoiding smoking for a day), supporting goal setting techniques. As users advance, tasks gradually become more difficult, exemplifying the use of *graded tasks* to encourage behavior change in manageable steps. Feedback and self-monitoring tools are also central to gamification, providing users with immediate, engaging updates such as progress bars, activity logs, or performance summaries [15,17]. These features align with BCTs such as *self-monitoring of behavior* and *feedback on outcomes* and often incorporate rewards such as badges for meeting goals.

One significant advantage of using gamification in BCIs is that it allows for a wider variety of BCTs to be incorporated into a single intervention function, effectively acting as a toolkit to enrich the behavioral intervention applied [118]. One example is incentivization, which aims to influence motivation and behavior through reward mechanisms. Without gamification, mere incentivization-based interventions often rely on a narrow subset of BCTs within the “reward and threat” cluster, typically *material incentive (behavior*) or *material reward (behavior*), such as vouchers or redeemable points. However, gamification facilitates the inclusion of a broader spectrum of BCTs within the same cluster, such as *social reward*, through badges or avatar upgrades, expanding the ways rewards can be delivered, moving beyond mere monetary incentives to include symbolic, social, and intrinsic motivators [119].

Empirical evidence supports this distinction. A review of gamified health apps found that the median number of BCTs implemented per app was 14, with some apps using up to 22 techniques [120], while nongamified digital interventions often include only a few techniques [121].

## Designing and Implementing Gamified Interventions in Insurance Settings: Opportunities and Challenges

The existing evidence suggests that gamification can positively impact health and well-being [122] by promoting healthy behaviors [14,123] and sustaining user engagement in personal informatics applications designed for the quantified-self movement [124]. Gamification leverages people’s desire for competition and social interaction, serving as a powerful catalyst for encouraging lifestyle changes [125]. These effects form the basis for understanding how gamified interventions may generate value for both policyholders and insurers.

### Value Creation for Insurers and Policyholders

**Figure 1.**
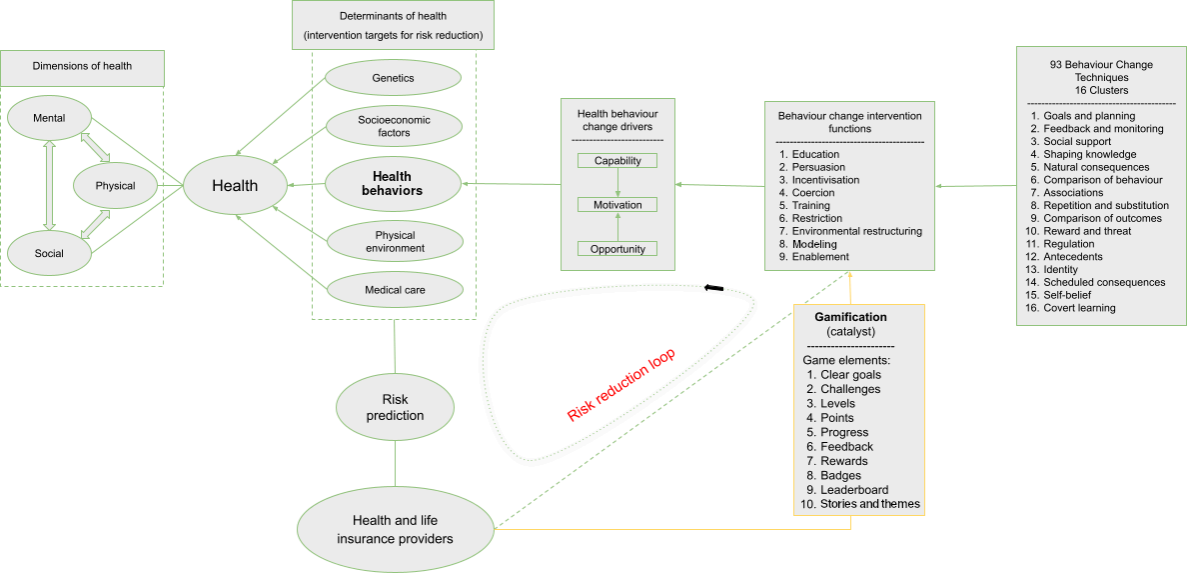
Overview of theories of behavioral change in health, their intersection with gamification, and the role of gamified interventions in supporting insurance providers’ risk reduction strategies.

The true power of gamification lies in the simultaneous use of multiple game elements. Figure 1 illustrates how gamification can enable the integration of BCTs into risk reduction initiatives and improve health outcomes (and thus insurer costs). A total of 93 BCTs comprise the foundational components of 9 broader intervention functions defined in the BCW framework. These functions influence the key drivers of behavior (COM-B system), which in turn shape individual health behaviors. These behaviors can be integrated into existing actuarial practices for more comprehensive risk assessment.

Additionally, as policyholders adopt healthier lifestyles, insurers experience lower health care claims, allowing them to offer reduced premiums and financial incentives. Similarly, engaged policyholders benefit from better health outcomes and lower insurance costs. While peer-reviewed studies in this area are lacking, a 5-year impact study of approximately 40,000 insured individuals reported that engaged members averaged US $462 less in annual medical claims, corresponding to a 4% reduction in claims costs with an estimated return on investment of approximately 180% [126].

Early evidence indicates that incentive-driven interventions can significantly boost engagement. Roughly two-thirds of consumers in a 2019 study said they would be willing to enroll in insurance wellness programs with wearables [127], with rates increasing further when rewards such as premium reductions were offered. While incentives can drive initial participation, gamified environments can increase user engagement further by helping satisfy basic psychological needs [128], such as facilitating a sense of community and support, and providing motivation and, thus, creating positive associations with the insurance provider. Such sustained engagement (often higher than industry standards [129,130]) can, in turn, help reduce customer lapse rates for insurance providers [131].

### Engagement, Data, and Personalization as Strategic Assets

High engagement through gamification provides insurers with strategic advantages, particularly richer data collection. Real-time behavioral data from gamified interactions can improve the accuracy of risk assessments, highlight emerging health risks before they manifest in claims, and enable personalized services, including dynamic premium pricing and tailored product offerings. Employers benefit from additional insights into the health of their employee population, enabling targeted wellness initiatives. Brokers can use behavioral profiles to identify optimal supplementary health products, enhancing cross-selling effectiveness. Reinsurers gain increased portfolio confidence, improving risk management and solvency assessments.

### Design and Implementation Challenges for Insurers

Designing effective products delivering gamified BCIs is a challenge. Gamification should strike a balance between extrinsic and intrinsic motivators, with intrinsic factors informed by theories of behavioral change, to ensure sustainable engagement and meaningful health improvements [132]. For insurers in the employer-sponsored group-cover market, any health gains arising from such interventions must be credibly reflected in underwriting, according to 3 core principles: risk pooling, actuarial fairness, and regulatory compliance, while also guarding against “2-tier market” selection effects [133], and implementing strict data-governance measures to build and maintain user trust [134]. Successfully implementing gamification strategies requires ongoing investment in advanced analytics, iterative product refinement based on user feedback, and collaborative integration across the broader insurance ecosystem to maximize collective benefits [135].

## Ethical and Practical Considerations of Gamification in Insurance Settings

Gamification in health insurance raises several ethical challenges. If engagement and behavioral data are analyzed to directly inform insurance policies, this can compromise people’s ability to engage with such risk-reducing interventions freely [38]. Interventions require design considerations and adequate monitoring to mitigate potential adverse effects (eg, on mental health or social well-being) [38,39]. This may be further problematic in employer-sponsored insurance, compromising autonomy and the ability to provide informed consent, particularly with regard to how personal behavioral data are handled [40]. In the absence of appropriate data agreements, function creep (ie, the use of data for purposes other than the original intent) could result in harm, for example, interpreting periods of illness as noncompliance or relying on out-of-scope data to counter insurance claims. Finally, inclusivity may be a concern, as standard game elements may not be equally accessible to all, resulting in a form of digital divide [136]. This could lead to adverse effects on insurance premiums and health outcomes in the affected populations, for example, through perceptions of nonadherence or indirect discrimination regarding rewards [41]. Therefore, ethical evaluation of gamified interventions should also address how data could be misused, rather than focus solely on privacy considerations. For a more detailed discussion on ethical and practical considerations, including practical and data security challenges, refer to Textbox S4 in Multimedia Appendix 1.

## Empirical Evidence From Systematic Reviews and Meta-Analyses

### Overview

While there is a sound theoretical basis for applying gamification in BCIs, empirical evidence addressing improvements in such initiatives should also be considered. However, studies evaluating gamification specifically within insurance settings are scarce. Therefore, to inform this viewpoint, we focused on evidence from broader digital health BCIs, which has been assessed in numerous health behavior change programs [15]. To inform this viewpoint, we draw on findings from published systematic reviews and meta-analyses examining the impact of gamification in BCIs, with a primary focus on PA (due to its prominence in the literature and relevance to population-level digital health and prevention strategies). Table 1 summarizes the key findings.

**Table 1.** Summary of systematic reviews and meta-analyses examining gamified behavior change interventions.

Study	Review type	Number of studies (participants)	Target population	Main findings
Mazeas et al [42] (2022)	Systematic review and meta-analysis	16 RCTs^a^ (n=2407)	General population (9‐73 y)	Small-to-moderate positive effect on PA^b^ (Hedges’ *g*=0.42; 95% CI 0.14‐0.69), step count increase of +1609 steps per day (95% CI 372-2847), and reduced long-term effect at follow-up (Hedges’ *g*=0.15)
Nishi et al [75] (2024)	Systematic review and meta-analysis	36 RCTs (n>10,000)	General adult populations	+489 steps per day (95% CI 64-914), BMI −0.28 kg/m², weight −0.70 kg, waist circumference −1.16 cm, and body fat −1.92%
Xu et al [76] (2022)	Systematic review	50 studies (n=9977)	Mixed populations (predominantly younger adults)	Mixed evidence and modest improvements in PA and engagement
Chen et al [77] (2025)	Systematic review	8 trials (n=1454)	Older adults (≥60 y)	Generally positive effects on steps and MVPA^c^, stronger effects in theory-driven and hybrid interventions, and effect sizes ranged from small to moderate
Wang et al [86] (2025)	Systematic review and meta-analysis	16 RCTs (n=7472)	Children and adolescents (6‐18 y)	Small increase in MVPA (standardized mean difference=0.15), BMI reduction (standardized mean difference=0.11), and no effect on steps or sedentary behavior
Alzghoul [88] (2024)	Systematic review and meta-analysis	30 RCTs (n=11,558)	Mixed populations	Significant pooled effect on behavior change (odds ratio 1.27, 95% CI 1.20-1.33) and no evidence of publication bias
Yu et al [89] (2025)	Systematic review and meta-analysis	6 RCTs (n=1109)	Adults with cardiovascular disease	Short-term PA effect (Hedges’ *g*=0.32), maintenance effect at follow-up (Hedges’ *g*=0.20), and +697 steps per day

a RCT: randomized controlled trial.

b PA: physical activity.

c MVPA: moderate-to-vigorous physical activity.

Overall, the studies summarized in Table 1 suggest that gamified interventions were generally associated with small-to-moderate PA improvements, mostly reflecting daily step counts, but also, to a lesser extent, “moderate-to-vigorous PA,” across diverse populations (eg, young, old, and chronically ill). However, individual studies vary considerably in terms of intervention design, outcome measures, and combinations of game elements, making comparisons across the spectrum somewhat challenging. This is further compounded by common methodological constraints, such as short interventions, limited follow-up, and challenges in isolating individual game component contributions. Brief summaries of individual reviews and additional references providing relevant context [78-85] are provided in Textbox S5 in Multimedia Appendix 1.

### Benefits and Limitations Identified in the Literature

Collectively, evidence from systematic reviews and meta-analyses indicates that gamified interventions are associated with positive effects on PA. Meta-analytic findings suggest small-to-moderate improvements in overall PA (eg, step count, moderate-to-vigorous PA, and active minutes), with statistically significant increases in daily step counts reported across several reviews. Beyond PA, additional benefits have been observed in other clinical and behavioral domains [137], including modest improvements in cardiometabolic risk markers [138], medication adherence [139], dietary behaviors [140,141], and short-term smoking abstinence [142]. Taken together, these findings suggest that gamification can successfully initiate behavior change and enhance engagement across diverse populations and health contexts.

However, a substantial proportion of studies report mixed or neutral outcomes. One key limitation is the dominance of extrinsic motivators, most notably financial incentives, which characterize many interventions labeled as gamified. While rewards are a legitimate game-design element and may take nonmonetary forms (eg, virtual items), the frequent coupling of gamification with direct financial incentives blurs the distinction between gamification and mere incentivization. This overlap makes it difficult to disentangle behavioral effects attributable to game mechanics from those driven primarily by monetary rewards, particularly when both are implemented concurrently.

Methodological limitations further constrain interpretation. Considerable heterogeneity in intervention design, target populations, and outcome measures, combined with the widespread use of multiple game elements simultaneously, limits the ability to isolate the effects of individual components and contributes to highly variable effect sizes across studies. Many trials are underpowered, rely on short intervention durations, or lack appropriate nongamified comparison groups, reducing confidence in causal attribution. Sustained effectiveness also remains a central challenge. Although short-term improvements are frequently observed, engagement and behavioral effects often diminish over time, with follow-up analyses consistently showing smaller and more volatile maintenance effects. Effectiveness also depends on alignment between game design and user characteristics. Features that motivate novice users may disengage more experienced users, while certain elements may be poorly suited to sensitive contexts such as mental health or mindfulness. Usability problems (such as complex interfaces or high interaction burden), declining engagement over time, privacy concerns, and limited personalization further constrain the scalability and long-term sustainability of gamified health interventions [15].

## Discussion

This paper explored the role of gamification in enhancing health BCIs, with particular emphasis on its application within the health and life insurance sector. We have highlighted the potential of gamification as a powerful tool for increasing engagement and promoting sustained behavior change. We showed that gamification enables the deployment and integration of multiple BCTs, targeting essential drivers of health behavior change. By mapping game elements to specific BCTs, designers can ensure that fun and engaging interactions also serve meaningful behavioral purposes. However, there are still some limitations to address. Gamification studies often report short-term outcomes but fail to address the sustainability of behavior change over time. As the novelty of gamified features wears off, maintaining long-term engagement becomes a challenge [14]. Moreover, many interventions lack appropriate control groups, making it difficult to isolate the specific contribution of gamification to the observed outcomes. Future research must prioritize long-term evaluations to investigate sustained behavior change and examine to what extent personalization can enhance gamification’s impact [143], as one-size-fits-all approaches may not be adequate in health contexts [144,145].

Practical opportunities for gamification extend beyond public health interventions and hold value for the health and life insurance sector. The increasing prevalence of chronic conditions, primarily driven by unhealthy behaviors such as sedentary lifestyles [146], places a growing financial burden on health care systems and insurance providers [147]. Gamification enables insurers to encourage healthier lifestyles among policyholders, potentially improving health outcomes and reducing claim costs. Moreover, gamification can enhance health literacy among younger adults [148], supporting better decision-making regarding insurance coverage and preventive behaviors.

One main finding emerging from this review is the lack of studies specific to the insurance sector, despite the emerging use of gamification in the sector. For insurers seeking to meet the varied needs and preferences of different demographics, leveraging a diverse range of game mechanics and BCTs, grounded in sound evidence, is essential. Programs must be inclusive, accessible, and continuously refined based on participant feedback and health outcomes. Establishing clear links between game elements, BCTs, and behavior change determinants can strengthen program evaluation and ensure interventions are underpinned by established behavioral science. In doing so, insurers encourage healthier behavior among policyholders and also create more sustainable, financially efficient insurance models.

## Conclusions

As digital health continues to advance, gamification emerges as a promising approach for enhancing health behavior interventions. When applied thoughtfully and evaluated rigorously, gamified strategies can significantly contribute to healthier populations, more proactive health care, and innovative service models in industries such as health and life insurance, having the potential to be a new panacea for the sector and for population health more generally. However, while gamification represents a powerful catalyst for advancing health behavior change, its application must be guided by careful, theory-based design and ongoing evaluation [149]. Ethical, practical, and regulatory considerations must also be considered to ensure that gamification is used responsibly. Looking ahead, the challenge lies not just in gamifying health, but in doing so in ways that are equitable, grounded in solid theoretical principles and evidence from the literature, and aligned with long-term public and individual well-being.

## Supplementary material

10.2196/80684 Multimedia Appendix 1 Tables and textboxes providing an overview of gamification in the UK insurance sector, core psychological theories of behavior change, ethical and practical considerations, evidence-based summaries from systematic reviews, and standardized taxonomies for behavior change interventions and techniques.

## Abbreviations

BCI: behavior change intervention
BCT: Behavior Change Technique
BCW: Behavior Change Wheel
COM-B: capability, opportunity, motivation, and behavior
NHS: National Health Service
PA: physical activity

## References

[1] (2017). Prevention before cure: securing the long-term sustainability of the NHS. British Medical Association.

[2] (2025). Healthcare expenditure, UK Health Accounts: 2023 and 2024. https://www.ons.gov.uk/peoplepopulationandcommunity/healthandsocialcare/healthcaresystem/bulletins/ukhealthaccounts/2023and2024.

[3] Strain T, Flaxman S, Guthold R (2024). National, regional, and global trends in insufficient physical activity among adults from 2000 to 2022: a pooled analysis of 507 population-based surveys with 5·7 million participants. Lancet Glob Health.

[4] (2022). Global status report on physical activity 2022. https://www.who.int/publications/i/item/9789240059153.

[5] Warburton DE, Bredin SS (2017). Health benefits of physical activity: a systematic review of current systematic reviews. Curr Opin Cardiol.

[6] San-Millan I, Martinez JL, Sparagna GC, D’Alessandro A, Stefanoni D, Nemkov T (2024). Metabolic and cellular differences between sedentary and active individuals at rest and during exercise. bioRxiv.

[7] Hernández-López OA, Murillo-Ortíz B, Luna-Marco C (2025). Cardiorespiratory fitness as a key predictor of metabolic, inflammatory, and oxidative stress biomarkers in adults with different physical activity levels. Free Radic Biol Med.

[8] (2022). Physical activity: applying All Our Health. Office for Health Improvement & Disparities, United Kingdom Government.

[9] Short SE, Mollborn S (2015). Social determinants and health behaviors: conceptual frames and empirical advances. Curr Opin Psychol.

[10] Kelly S, Martin S, Kuhn I, Cowan A, Brayne C, Lafortune L (2016). Barriers and facilitators to the uptake and maintenance of healthy behaviours by people at mid-life: a rapid systematic review. PLoS One.

[11] Marteau TM, Hollands GJ, Fletcher PC (2012). Changing human behavior to prevent disease: the importance of targeting automatic processes. Science.

[12] Schlaff AL (2013). Behavior change in America: public health, medicine, and individual counseling. Virtual Mentor.

[13] Deterding S, Dixon D, Khaled R, Nacke L From game design elements to gamefulness: defining “gamification”.

[14] Cugelman B (2013). Gamification: what it is and why it matters to digital health behavior change developers. JMIR Serious Games.

[15] Johnson D, Deterding S, Kuhn KA, Staneva A, Stoyanov S, Hides L (2016). Gamification for health and wellbeing: a systematic review of the literature. Internet Interv.

[16] Uechi H, Tan N, Honda Y (2018). Effects of gamification-based intervention for promoting health behaviors. J Phys Fitness Sports Med.

[17] Lister C, West JH, Cannon B, Sax T, Brodegard D (2014). Just a fad? Gamification in health and fitness apps. JMIR Serious Games.

[18] Hamari J, Koivisto J Social motivations to use gamification: an empirical study of gamifying exercise. https://www.researchgate.net/publication/236269293_Social_motivations_to_use_gamification_An_empirical_study_of_gamifying_exercise.

[19] Hamari J, Koivisto J (2015). “Working out for likes”: an empirical study on social influence in exercise gamification. Comput Human Behav.

[20] Wing RR, Jeffery RW (1999). Benefits of recruiting participants with friends and increasing social support for weight loss and maintenance. J Consult Clin Psychol.

[21] Graupensperger S, Gottschall JS, Benson AJ, Eys M, Hastings B, Evans MB (2019). Perceptions of groupness during fitness classes positively predict recalled perceptions of exertion, enjoyment, and affective valence: an intensive longitudinal investigation. Sport Exerc Perform Psychol.

[22] Fanaroff AC, Patel MS, Chokshi N (2024). Effect of gamification, financial incentives, or both to increase physical activity among patients at high risk of cardiovascular events: the BE ACTIVE randomized controlled trial. Circulation.

[23] Giles EL, Robalino S, McColl E, Sniehotta FF, Adams J (2014). The effectiveness of financial incentives for health behaviour change: systematic review and meta-analysis. PLoS One.

[24] Promberger M, Marteau TM (2013). When do financial incentives reduce intrinsic motivation? Comparing behaviors studied in psychological and economic literatures. Health Psychol.

[25] Dahlstrøm C (2017). Impacts of gamification on intrinsic motivation [Master’s thesis]. https://www.ntnu.edu/documents/139799/1279149990/04%2BArticle%2BFinal_camildah_fors%25C3%25B8k_2017-12-06-13-53-55_TPD4505.Camilla.Dahlstr%25C3%25B8m.pdf.

[26] Sardi L, Idri A, Fernández-Alemán JL (2017). A systematic review of gamification in e-Health. J Biomed Inform.

[27] Bardach L, Murayama K (2025). The role of rewards in motivation—beyond dichotomies. Learn Instr.

[28] Wongvibulsin S, Martin SS, Saria S, Zeger SL, Murphy SA (2020). An individualized, data-driven digital approach for precision behavior change. Am J Lifestyle Med.

[29] Glanz K, Rimer BK, Viswanath K (2008). Health Behavior and Health Education: Theory, Research, and Practice.

[30] Zhang X, Gao P, Snyder MP (2021). The exposome in the era of the quantified self. Annu Rev Biomed Data Sci.

[31] Swan M (2013). The quantified self: fundamental disruption in big data science and biological discovery. Big Data.

[32] Ossenbrink L, Haase T, Timpel P (2023). Effectiveness of digital health interventions containing game components for the self-management of type 2 diabetes: systematic review. JMIR Serious Games.

[33] Chimatapu SN, Mittelman SD, Habib M, Osuna-Garcia A, Vidmar AP (2024). Wearable devices beyond activity trackers in youth with obesity: summary of options. Child Obes.

[34] Murray E, Hekler EB, Andersson G (2016). Evaluating digital health interventions: key questions and approaches. Am J Prev Med.

[35] Son YS, Kwon KH (2024). Utilization of smart devices and the evolution of customized healthcare services focusing on big data: a systematic review. Mhealth.

[36] Gamification market size & share analysis - growth trends and forecast (2026 - 2031). Mordor Intelligence.

[37] Ryan RM, Deci EL (2000). Intrinsic and extrinsic motivations: classic definitions and new directions. Contemp Educ Psychol.

[38] Kim TW, Werbach K (2016). More than just a game: ethical issues in gamification. Ethics Inf Technol.

[39] Yang H, Li D (2021). Understanding the dark side of gamification health management: a stress perspective. Inf Process Manag.

[40] Durieux BN, DeCamp M, Lindvall C (2022). 21st Century Cures Act: ethical recommendations for new patient-facing products. J Am Med Inform Assoc.

[41] Arora C, Razavian M (2021). Ethics of gamification in health and fitness-tracking. Int J Environ Res Public Health.

[42] Mazeas A, Duclos M, Pereira B, Chalabaev A (2022). Evaluating the effectiveness of gamification on physical activity: systematic review and meta-analysis of randomized controlled trials. J Med Internet Res.

[43] Rewards partners. Vitality.

[44] Vitality to transform client engagement with evolution of vitality programme with launch of pick and play. Vitality.

[45] (2025). From steps to science: how gamification is transforming health and life insurance. Yu Life.

[46] (2024). YuLife partners with MetLife UK to launch pre-early intervention group income protection solution. Yu Life.

[47] (2023). YuLife launches group health insurance provided by Bupa: here’s how it works. Yu Life.

[48] (2025). Does gamification still work, or is it a trend from the past?. Yu Life.

[49] Business health insurance. Aviva.

[50] Find out everything your AXA health membership offers you. AXA Health.

[51] Alyafei A, Easton-Carr R (2024). StatPearls.

[52] Rosenstock IM, Strecher VJ, Becker MH (1988). Social learning theory and the Health Belief Model. Health Educ Q.

[53] Holloway A, Watson HE (2002). Role of self-efficacy and behaviour change. Int J Nurs Pract.

[54] Schwarzer R, Warner LM, Prince-Embury S, Saklofske DH (2013). Resilience in Children, Adolescents, and Adults: Translating Research Into Practice.

[55] French DP, Olander EK, Chisholm A, Mc Sharry J (2014). Which behaviour change techniques are most effective at increasing older adults’ self-efficacy and physical activity behaviour? A systematic review. Ann Behav Med.

[56] Prestwich A, Kellar I, Parker R (2014). How can self-efficacy be increased? Meta-analysis of dietary interventions. Health Psychol Rev.

[57] Olander EK, Fletcher H, Williams S, Atkinson L, Turner A, French DP (2013). What are the most effective techniques in changing obese individuals’ physical activity self-efficacy and behaviour: a systematic review and meta-analysis. Int J Behav Nutr Phys Act.

[58] Newby K, Teah G, Cooke R (2021). Do automated digital health behaviour change interventions have a positive effect on self-efficacy? A systematic review and meta-analysis. Health Psychol Rev.

[59] Ashford S, Edmunds J, French DP (2010). What is the best way to change self-efficacy to promote lifestyle and recreational physical activity? A systematic review with meta-analysis. Br J Health Psychol.

[60] Ryan RM, Deci EL (2000). Self-determination theory and the facilitation of intrinsic motivation, social development, and well-being. Am Psychol.

[61] Ng JY, Ntoumanis N, Thøgersen-Ntoumani C (2012). Self-determination theory applied to health contexts: a meta-analysis. Perspect Psychol Sci.

[62] Fogg BJ A behavior model for persuasive design.

[63] Ryan RM (1995). Psychological needs and the facilitation of integrative processes. J Pers.

[64] Deci EL (2012). Intrinsic Motivation.

[65] Seligman ME (1975). Helplessness: On Depression, Development, and Death.

[66] Ntoumanis N, Ng JY, Prestwich A (2021). A meta-analysis of self-determination theory-informed intervention studies in the health domain: effects on motivation, health behavior, physical, and psychological health. Health Psychol Rev.

[67] Oudeyer PY, Kaplan F (2007). What is intrinsic motivation? A typology of computational approaches. Front Neurorobot.

[68] (2010). Equality Act 2010. legislation.gov.uk.

[69] What are the rules on special category data?. Information Commisioner’s Office.

[70] What are the conditions for processing?. Information Commissioner’s Office.

[71] A guide to the data protection principles. Information Commissioner’s Office.

[72] PS22/9: a new consumer duty. Financial Conduct Authority.

[73] Automated decision-making, including profiling. Information Commissioner’s Office.

[74] Explaining decisions made with AI. Information Commissioner’s Office.

[75] Nishi SK, Kavanagh ME, Ramboanga K (2024). Effect of digital health applications with or without gamification on physical activity and cardiometabolic risk factors: a systematic review and meta-analysis of randomized controlled trials. EClinicalMedicine.

[76] Xu L, Shi H, Shen M (2022). The effects of mHealth-based gamification interventions on participation in physical activity: systematic review. JMIR Mhealth Uhealth.

[77] Chen L, Jang F, Li M, Zong W, Yu H (2025). Effectiveness of mHealth-based gamified interventions on physical activity in older adults: systematic review. JMIR Aging.

[78] Kahneman D, Tversky A, MacLean LC, Ziemba WT (2013). Handbook of the Fundamentals of Financial Decision Making: Part I.

[79] Greysen SR, Oon AL, Harkins K (2024). Effect of gamification with a support partner to increase physical activity in older adults at risk for Alzheimer’s disease: the STEP 4Life randomized clinical trial. Alzheimers Dement.

[80] Martinho D, Crista V, Carneiro J, Matsui K, Corchado JM, Marreiros G (2023). Effects of a gamified agent-based system for personalized elderly care: pilot usability study. JMIR Serious Games.

[81] Santos LH, Okamoto K, Hiragi S (2019). Pervasive game design to evaluate social interaction effects on levels of physical activity among older adults. J Rehabil Assist Technol Eng.

[82] Santos LH, Okamoto K, Funghetto SS (2019). Effects of social interaction mechanics in pervasive games on the physical activity levels of older adults: quasi-experimental study. JMIR Serious Games.

[83] Santos LH, Okamoto K, Otsuki R (2021). Promoting physical activity in Japanese older adults using a social pervasive game: randomized controlled trial. JMIR Serious Games.

[84] Randriambelonoro M, Perrin Franck C, Herrmann F (2023). Gamified physical rehabilitation for older adults with musculoskeletal issues: pilot noninferiority randomized clinical trial. JMIR Rehabil Assist Technol.

[85] Kawaguchi K, Nakagomi A, Ide K, Kondo K (2024). Effects of a mobile app to promote social participation on older adults: randomized controlled trial. J Med Internet Res.

[86] Wang M, Xu J, Zhou X, Li X, Zheng Y (2025). Effectiveness of gamification interventions to improve physical activity and sedentary behavior in children and adolescents: systematic review and meta-analysis. JMIR Serious Games.

[87] Michie S, van Stralen MM, West R (2011). The behaviour change wheel: a new method for characterising and designing behaviour change interventions. Implement Sci.

[88] Alzghoul B (2024). The effectiveness of gamification in changing health-related behaviors: a systematic review and meta-analysis. Open Public Health J.

[89] Yu T, Parry M, Yu T (2025). Effectiveness of mobile health-based gamification interventions for improving physical activity in individuals with cardiovascular diseases: systematic review and meta-analysis of randomized controlled trials. JMIR Serious Games.

[90] Michie S, Richardson M, Johnston M (2013). The behavior change technique taxonomy (v1) of 93 hierarchically clustered techniques: building an international consensus for the reporting of behavior change interventions. Ann Behav Med.

[91] Denecke K, Schmid N, Nüssli S (2022). Implementation of cognitive behavioral therapy in e-mental health apps: literature review. J Med Internet Res.

[92] Timmers T, Janssen L, Kool RB, Kremer JA (2020). Educating patients by providing timely information using smartphone and tablet apps: systematic review. J Med Internet Res.

[93] Pérez-Jover V, Sala-González M, Guilabert M, Mira JJ (2019). Mobile apps for increasing treatment adherence: systematic review. J Med Internet Res.

[94] Maier M, Carlotto H, Sanchez F, Balogun S, Merritt S (2019). Transforming underwriting in the life insurance industry. Proc AAAI Conf Artif Intell.

[95] Brackenridge RD, Croxson RS, Mackenzie R (2016). Brackenridge's Medical Selection of Life Risks.

[96] Castaneda MA, Marton J (2013). Employer-provided health insurance and the adverse selection problem. Public Finan Rev.

[97] Malali N (2025). Artificial intelligence in life insurance underwriting: a risk assessment and ethical implications. Int J Interdiscip Res Methods.

[98] Kang HS, Exworthy M (2022). Wearing the future-wearables to empower users to take greater responsibility for their health and care: scoping review. JMIR Mhealth Uhealth.

[99] (2024). The UK Group Risk industry paid out a record £2.49bn in claims in 2023: £6.82m a day. GRiD.

[100] Dah J, Hussin N, Zaini MK, Isaac Helda L, Senanu Ametefe D, Adozuka Aliu A (2025). Gamification is not working: why?. Games Cult.

[101] Manzoni JF, Epstein MJ, Manzoni JF, Davila A (2010). Performance Measurement and Management Control: Innovative Concepts and Practices.

[102] Davis R, Campbell R, Hildon Z, Hobbs L, Michie S (2015). Theories of behaviour and behaviour change across the social and behavioural sciences: a scoping review. Health Psychol Rev.

[103] Michie S, Prestwich A (2010). Are interventions theory-based? Development of a theory coding scheme. Health Psychol.

[104] Stevely AK, Buykx P, Brown J (2018). Exposure to revised drinking guidelines and “COM-B” determinants of behaviour change: descriptive analysis of a monthly cross-sectional survey in England. BMC Public Health.

[105] (2019). Achieving behaviour change: a guide for local government and partners. https://assets.publishing.service.gov.uk/media/5e7b4e85d3bf7f133c923435/PHEBI_Achieving_Behaviour_Change_Local_Government.pdf.

[106] (2023). Evidence-based intervention design for behaviour change during a health emergency. UNICEF.

[107] Power S, Broom D, Duncan M, Biddle S, Rowley N (2024). Using the behavior change wheel to design a novel home-based exercise program for adults living with overweight and obesity: comprehensive reporting of intervention development. Obes Sci Pract.

[108] Ojo SO, Bailey DP, Brierley ML, Hewson DJ, Chater AM (2019). Breaking barriers: using the behavior change wheel to develop a tailored intervention to overcome workplace inhibitors to breaking up sitting time. BMC Public Health.

[109] Meloncelli N, O’Connor H, de Jersey S (2024). Designing a behaviour change intervention using COM‐B and the Behaviour Change Wheel: co‐designing the Healthy Gut Diet for preventing gestational diabetes. J Hum Nutr Diet.

[110] Reid H, Smith R, Williamson W (2022). Use of the behaviour change wheel to improve everyday person-centred conversations on physical activity across healthcare. BMC Public Health.

[111] Craig P, Dieppe P, Macintyre S (2008). Developing and evaluating complex interventions: the new Medical Research Council guidance. BMJ.

[112] Michie S, Abraham C, Eccles MP, Francis JJ, Hardeman W, Johnston M (2011). Strengthening evaluation and implementation by specifying components of behaviour change interventions: a study protocol. Implement Sci.

[113] Marques MM, Wright AJ, Corker E (2023). The behaviour change technique ontology: transforming the Behaviour Change Technique Taxonomy v1. Wellcome Open Res.

[114] Michie S, Carey RN, Johnston M (2018). From theory-inspired to theory-based interventions: a protocol for developing and testing a methodology for linking behaviour change techniques to theoretical mechanisms of action. Ann Behav Med.

[115] Young MD, Collins CE, Callister R, Plotnikoff RC, Doran CM, Morgan PJ (2014). The SHED-IT weight loss maintenance trial protocol: a randomised controlled trial of a weight loss maintenance program for overweight and obese men. Contemp Clin Trials.

[116] Po’e EK, Heerman WJ, Mistry RS, Barkin SL (2013). Growing Right Onto Wellness (GROW): a family-centered, community-based obesity prevention randomized controlled trial for preschool child-parent pairs. Contemp Clin Trials.

[117] Balci S, Secaur JM, Morris BJ (2022). Comparing the effectiveness of badges and leaderboards on academic performance and motivation of students in fully versus partially gamified online physics classes. Educ Inf Technol (Dordr).

[118] Milne-Ives M, Homer SR, Andrade J, Meinert E (2023). Potential associations between behavior change techniques and engagement with mobile health apps: a systematic review. Front Psychol.

[119] Kurtzman GW, Day SC, Small DS (2018). Social incentives and gamification to promote weight loss: the LOSE IT randomized, controlled trial. J Gen Intern Med.

[120] Edwards EA, Lumsden J, Rivas C (2016). Gamification for health promotion: systematic review of behaviour change techniques in smartphone apps. BMJ Open.

[121] Middelweerd A, Mollee JS, van der Wal CN, Brug J, Te Velde SJ (2014). Apps to promote physical activity among adults: a review and content analysis. Int J Behav Nutr Phys Act.

[122] Litvin S, Saunders R, Maier MA, Lüttke S (2020). Gamification as an approach to improve resilience and reduce attrition in mobile mental health interventions: a randomized controlled trial. PLoS One.

[123] Czerska I (2024). Gamification in health promotion in the context of creating the concept of Health 2.0 - a comparative analysis of selected health mobile applications. Eur Res Stud J.

[124] Morschheuser BS, Rivera-Pelayo V, Mazarakis A, Zacharias V (2014). Interaction and reflection with quantified self and gamification: an experimental study. J Lit Technol.

[125] Patel MS, Small DS, Harrison JD (2021). Effect of behaviorally designed gamification with social incentives on lifestyle modification among adults with uncontrolled diabetes: a randomized clinical trial. JAMA Netw Open.

[126] (2024). Impact study. Vitality Group.

[127] Soliño-Fernandez D, Ding A, Bayro-Kaiser E, Ding EL (2019). Willingness to adopt wearable devices with behavioral and economic incentives by health insurance wellness programs: results of a US cross-sectional survey with multiple consumer health vignettes. BMC Public Health.

[128] Bitrián P, Buil I, Catalán S (2021). Enhancing user engagement: the role of gamification in mobile apps. J Bus Res.

[129] Roomer J (2022). How YuLife uses game mechanics to drive 3x the average benefits engagement rates. YuLife.

[130] (2022). Engagement white paper. Vitality.

[131] Adviser FAQs. AIA Australia.

[132] Seifert CM, Chapman LS, Hart JK, Perez P (2012). Enhancing intrinsic motivation in health promotion and wellness. Am J Health Promot.

[133] Rothschild C, Thistle PD (2022). Supply, demand, and selection in insurance markets: theory and applications in pictures. Risk Manag Insur Rev.

[134] Ho CW, Ali J, Caals K (2020). Ensuring trustworthy use of artificial intelligence and big data analytics in health insurance. Bull World Health Organ.

[135] Chen Y (2019). Exploring design guidelines of using user-centered design in gamification development: a delphi study. Int J Hum Comput Interact.

[136] Lythreatis S, Singh SK, El-Kassar AN (2022). The digital divide: a review and future research agenda. Technol Forecast Soc Change.

[137] Yau KW, Tang TS, Görges M (2022). Effectiveness of mobile apps in promoting healthy behavior changes and preventing obesity in children: systematic review. JMIR Pediatr Parent.

[138] Mitra S, Kroeger CM, Wang T (2024). The impact of gamified smartphone app interventions on behaviour and metabolic outcomes in individuals at risk of cardiovascular disease. Stud Health Technol Inform.

[139] Tran S, Smith L, El-Den S, Carter S (2022). The use of gamification and incentives in mobile health apps to improve medication adherence: scoping review. JMIR Mhealth Uhealth.

[140] Yoshida-Montezuma Y, Ahmed M, Ezezika O (2020). Does gamification improve fruit and vegetable intake in adolescents? A systematic review. Nutr Health.

[141] Suleiman-Martos N, García-Lara RA, Martos-Cabrera MB (2021). Gamification for the improvement of diet, nutritional habits, and body composition in children and adolescents: a systematic review and meta-analysis. Nutrients.

[142] Rajani NB, Bustamante L, Weth D, Romo L, Mastellos N, Filippidis FT (2023). Engagement with gamification elements in a smoking cessation app and short-term smoking abstinence: quantitative assessment. JMIR Serious Games.

[143] Nurmi J, Knittle K, Ginchev T (2020). Engaging users in the behavior change process with digitalized motivational interviewing and gamification: development and feasibility testing of the precious app. JMIR Mhealth Uhealth.

[144] Cheng VW, Davenport T, Johnson D, Vella K, Hickie IB (2019). Gamification in apps and technologies for improving mental health and well-being: systematic review. JMIR Ment Health.

[145] Cheng VWS (2020). Recommendations for implementing gamification for mental health and wellbeing. Front Psychol.

[146] Huston P (2022). A sedentary and unhealthy lifestyle fuels chronic disease progression by changing interstitial cell behaviour: a network analysis. Front Physiol.

[147] Heron L, O’Neill C, McAneney H, Kee F, Tully MA (2019). Direct healthcare costs of sedentary behaviour in the UK. J Epidemiol Community Health.

[148] Zikra AA (2023). The impact of gamified interventions on youth health literacy: a systematic review of effectiveness and implementation challenges. JRKPK.

[149] Schmidt-Kraepelin M, Warsinsky S, Thiebes S, Sunyaev A The role of gamification in health behavior change: a review of theory-driven studies.

