# Supplementary material for: Is Gamification the New Panacea for Health Behavioral Changes? Implications for the Health and Life Insurance Industry
Source: JMIR Serious Games. 2026 Apr 13;14:e80684. doi: 10.2196/80684 (PMC13075540; doi:10.2196/80684)
Supplement: Multimedia Appendix 1 [file games-v14-e80684-s001.docx]

Multimedia Appendix 1: Tables and Boxes

This is a Multimedia Appendix to a full manuscript published in the J Med Internet Res, entitled “Is gamification the new panacea for health-behavioral changes? Implications for the Health and Life Insurance Industry.” by A. Salami et al. For full copyright and citation information, see <http://dx.doi.org/10.2196/80684>

Table of contents

[Box 1 – Use of gamification in the UK Health and Life insurance sector](#_y4yo3skfemm3)

[Box 2 – Core Psychological Theories and Constructs of Behavioral Change](#_a6167r4wi7hi)

[Health Belief Model (HBM)](#_f4z26ulwmfb)

[Self-efficacy](#_iqnccfcdh2wv)

[Self-Determination Theory (SDT)](#_6v1d5yai4ehm)

[Fogg Behavior Model (FBM)](#_ck85ovq4yep0)

[Box 3 – Key Drivers of Health Behavior Change in the COM-B System: Definitions and Examples](#_qx7igso6depr)

[Box 4 – Ethical and Practical Considerations of gamification in the context of health insurance](#_d1l1wneaf8uc)

[Ethical issues in gamification.](#_dd7vb7x6gb2h)

[Practical and data security challenges.](#_l7wjdz70no7w)

[Box 5 – Evidence from Systematic Reviews and Meta-Analyses: approach and brief summaries](#_86madgfelc5b)

[Table 1 – Definitions of the behavior change intervention functions defined in the BCW [54]](#_645k0jliya6p)

[Table 2 – Summary of the 16 Behavior Change Technique (BCT) clusters as defined in BCT Taxonomy v1 [55]](#_huk68bpj4uou)

##

## Box 1 – Use of gamification in the UK Health and Life insurance sector

The adoption of gamification in the UK health and life insurance sector varies significantly. Some companies are leading the way with comprehensive programs, while others are integrating a few game elements as part of broader wellness initiatives or gamification through strategic partnerships. Vitality offers an app-based program that rewards users with points for engaging in PAs [[1]](https://paperpile.com/c/hIfrS0/azS2A). These points unlock weekly Active Rewards, such as Amazon vouchers, cinema tickets, or discounted Apple Watches. By earning Vitality points, users can progress through a status-based system (from Bronze to Platinum) to access additional benefits, including reduced excess payments and lower renewal premiums. The recently launched "Pick and Play" feature enhances the gamified experience by allowing members who achieve a certain number of activity points each week to play a game in search of a hidden mascot for extra rewards [[2]](https://paperpile.com/c/hIfrS0/1vvI). Another innovator in this space is YuLife, which incorporates a variety of game elements into its award-winning mobile app, designed as a health behavior intervention tool [[3]](https://paperpile.com/c/hIfrS0/2P2xl). Users of the YuLife app (acquired either directly or through strategic partnerships with insurance companies like MetLife [[4]](https://paperpile.com/c/hIfrS0/T2N0e) and Bupa [[5]](https://paperpile.com/c/hIfrS0/Mepfm)) can complete daily wellness quests, such as walking or meditating, to earn YuCoin, the app's virtual currency. This currency can be exchanged for high-street vouchers or used for donations. The YuLife app immerses users in a game-like "Yuniverse" filled with themed virtual environments, including social challenges and digital leaderboards that enable competition (e.g., through step "duels") and allow users to track their progress relative to peers [[6]](https://paperpile.com/c/hIfrS0/8jtcZ). Other companies in the UK, such as Aviva (with the Aviva Wellbeing App [[7]](https://paperpile.com/c/hIfrS0/JtAoB)) and AXA Health [[8]](https://paperpile.com/c/hIfrS0/NnT7M), also offer programs that use similar point systems to encourage customers and policyholders to participate in wellness activities. Some of these programs allow users to compete with their peers in achieving health goals and earn additional prizes.

####

## Box 2 – Core Psychological Theories and Constructs of Behavioral Change

### Health Belief Model (HBM)

The Health Belief Model (HBM) [[9]](https://paperpile.com/c/hIfrS0/Vd83s) is one of the earliest theoretical, predictive models of health-behavior, providing guidance and insight into how health-promotion programs fail. HBM is based on the theory that individuals' health-related actions are influenced by the fear of (developing) severe illnesses and the perceived fear-reducing benefits associated with recommended behavioral changes. People weigh the expected advantages of altering their behavior against the practical and psychological impediments before deciding to take action. HBM was later expanded to incorporate self-efficacy, recognizing that perceptions of competence are essential for translating intention into action [[10]](https://paperpile.com/c/hIfrS0/bIIxh).

### Self-efficacy

Self-efficacy, defined as an individual's beliefs about their ability to perform certain behaviors, is a critical construct in many health-behavior theories [[11]](https://paperpile.com/c/hIfrS0/wHYU8). Self-efficacy beliefs are cognitive factors that are critical in initiating health-behavior changes, determining the effort exerted in the process, and whether one perseveres in the face of obstacles [[12]](https://paperpile.com/c/hIfrS0/sJaIP). Evidence supports its central role in digital and behavioral interventions [[13–16]](https://paperpile.com/c/hIfrS0/qge7P+uwg7p+6JmBf+iMFrN). A meta-analysis of automated digital health-interventions showed small but positive effects on self-efficacy across behaviors such as healthy eating, PA, safer sexual behaviors, and smoking-cessation [[16]](https://paperpile.com/c/hIfrS0/iMFrN). A separate meta-analysis found that PA interventions led to significant improvements in self-efficacy, particularly when including feedback on performance and vicarious experience [[17]](https://paperpile.com/c/hIfrS0/YpEyb).

While HBM and self-efficacy focus primarily on cognitive evaluations and confidence, they say less about the quality of motivation that sustains behavior over time. Self-Determination Theory (SDT) addresses this gap.

### Self-Determination Theory (SDT)

SDT shifts attention from risk appraisal and confidence to the type of motivation driving behavior [[18]](https://paperpile.com/c/hIfrS0/X3bF0). SDT proposes that long-term behavior-change is more likely when three basic psychological needs are supported: autonomy (feeling that one's actions are self-chosen rather than imposed), competence (feeling capable of successfully performing the behavior), and relatedness (feeling socially connected and supported). The theory distinguishes autonomous motivation (acting in alignment with personal values or interests) from controlled motivation (acting due to pressure or external incentives) [[19]](https://paperpile.com/c/hIfrS0/EmTrn).

However, even when motivation is high and self-efficacy is strong, individuals may still fail to act in real-world contexts. This gap between internal drive and immediate action is explained by the Fogg Behavior Model (FBM).

### Fogg Behavior Model (FBM)

FBM states that action occurs only when Motivation, Ability, and a Prompt converge (“*B=MAP”*) [[20]](https://paperpile.com/c/hIfrS0/3Jyqy). This highlights that even highly-motivated individuals may fail to act if the behavior is too difficult (low ability) or if no prompt is delivered; equally, a perfectly-timed prompt will not work if motivation or ability is insufficient. FBM is particularly valuable for designing interventions, as it focuses on the practical mechanics: ensuring the individual is prompted at the right moment and that the required effort (ability) is minimized. Conceptually, FBM's "ability" component aligns with both self-efficacy and HBM's "perceived barriers", while the "prompt" mechanism mirrors HBM's "cues to action".

## Box 3 – Key Drivers of Health Behavior Change in the COM-B System: Definitions and Examples

*Capability* denotes an individual's psychological and physical capacity to engage in a behavior, including the necessary knowledge and skills to perform it. Physical capability specifically pertains to bodily functions, encompassing factors like strength, stamina, or the dexterity necessary for performing a behavior. For example, completing a 30-minute workout requires adequate physical capability, which may be hindered in individuals with joint disorders. Psychological capability, on the other hand, pertains to the cognitive capacity for necessary thought processes, including comprehension and reasoning. For instance, a person wants to start a healthy meal-planning habit but struggles because they find nutritional information confusing.

*Opportunity* is understood as all external factors that enable or prompt a behavior. This component can be further categorized into physical and social dimensions. The physical dimension refers to the environmental setting in which the behavior occurs, while the social dimension refers to norms and cues within the social domain that may encourage or discourage certain behaviors. For example, individuals living in deprived areas may lack physical opportunities to engage in healthy behaviors due to limited access to gyms, safe streets, or recreational facilities. Additionally, in some societies, cultural norms or social restrictions can make it uncomfortable for certain groups within the population, such as women, to exercise in public, thereby discouraging activities like cycling or jogging.

*Motivation* is a key component within the "COM-B" system, characterized as the driving force that initiates, directs, and maintains behaviors. Within the taxonomies of motivation, three distinct types are typically identified [[21]](https://paperpile.com/c/hIfrS0/VnpyQ): amotivation, intrinsic motivation, and extrinsic motivation. Amotivation describes a state characterized by a lack of intention to act, arising from a failure to appreciate an activity [[22]](https://paperpile.com/c/hIfrS0/T3vJI), a perceived lack of competence to engage in it [[23]](https://paperpile.com/c/hIfrS0/t8YlN), or a belief that the activity will not yield desired outcomes [[24]](https://paperpile.com/c/hIfrS0/md1J7). Intrinsic motivation signifies engagement in behavior driven by enjoyment and personal interest [[25]](https://paperpile.com/c/hIfrS0/roA83). It refers to pursuing an activity for its inherent satisfaction rather than for any external consequences [[26]](https://paperpile.com/c/hIfrS0/rhxuJ). When intrinsically motivated, individuals tend to act for the enjoyment or challenges involved rather than due to external pressures, rewards, or outcomes. Conversely, extrinsic motivation is fueled by external factors, such as the pursuit of financial rewards or the avoidance of punishment. In the COM-B system, which lies at the center of the BCW framework, each of these three components can have a reciprocal impact on human behaviors, while capability and opportunity also have the potential to impact motivation.

## Box 4 – Ethical and Practical Considerations of gamification in the context of health insurance

### Ethical issues in gamification.

Gamification raises ethical challenges that are amplified in health and life insurance because engagement and behavioral data can affect decisions with direct financial consequences. The core ethical considerations for using gamification in BCIs revolve around consumer welfare and autonomy, emphasizing the importance of ensuring that gamified interventions do not manipulate or coerce individuals in ways that compromise their ability to make free and informed choices [[27]](https://paperpile.com/c/hIfrS0/rmADG). Consequently, the ethical landscape necessitates careful consideration of intended and unintended behavioral outcomes. While the primary objective is to encourage positive behavior change, monitoring and mitigating potential adverse effects on individuals' mental health or social wellbeing is critical [[27]](https://paperpile.com/c/hIfrS0/rmADG). From a practical standpoint, this requires leveraging game mechanics such as points, badges, and leaderboards in a manner that encourages healthy behaviors without inducing stress or competition anxiety [[28]](https://paperpile.com/c/hIfrS0/5HCxP).

In insurance settings, however, autonomy and voluntariness may be reduced by financial incentives, loss-framed rewards, or employer-sponsored participation. Consent must therefore be robust, covering what data are collected, how they are interpreted (including profiling), and whether participation or non-participation can affect material terms such as rewards, renewals, or premiums [[29]](https://paperpile.com/c/hIfrS0/JltIZ).

Furthermore, inclusivity is essential to avoid embedding inequity in program design [[30]](https://paperpile.com/c/hIfrS0/cyq12). Standard metrics (e.g., step targets, streaks, leaderboards) may disadvantage people with disabilities, chronic conditions, older adults, or those with constrained opportunities to participate, creating indirect discrimination if linked to pricing, eligibility, exclusions, or claim scrutiny [[31]](https://paperpile.com/c/hIfrS0/xUQN0). These risks are compounded by measurement error and missingness in wearable and app data (e.g., device non-wear, sensor inaccuracy, intermittent connectivity), which can be misread as non-adherence or elevated risk.

A key insurance-specific concern arising from these data limitations is downstream use (function creep), whereby wellness data is repurposed for underwriting, renewal pricing, exclusions, fraud investigations, or claims assessment. Two illustrative misuse scenarios include: (i) activity declines during illness, injury, or caring responsibilities are treated as risk-relevant non-compliance, resulting in worse renewal terms; and (ii) historic activity, sleep, or location logs are requested during a claim and interpreted as evidence against symptom onset, impairment severity, or disclosure. These scenarios highlight why ethical evaluation must address how wellness data could be used to deny claims or impose unfair rates, rather than focusing solely on privacy at the point of collection.

### Practical and data security challenges.

Because insurer-led gamified programs would routinely process health-related information, the governance of such programs must prioritize privacy, transparency, and accountability. Under the UK GDPR and the Data Protection Act 2018, wearable-derived data may constitute special category data and requires a clear lawful basis, purpose limitation, data minimization, and appropriate safeguards [[32–34]](https://paperpile.com/c/hIfrS0/o1eGE+N0ojm+TEcJK). Complementing these data standards, the FCA Consumer Duty reinforces expectations of consumer understanding, fair value, and the avoidance of foreseeable harm, especially for vulnerable customers [[35]](https://paperpile.com/c/hIfrS0/n6uGZ). Where behavioral data informs decisions with legal or similarly significant effects (e.g., premium changes or claim outcomes), safeguards related to profiling and automated decision-making are central, including meaningful information, contestability, and access to human review [[36,37]](https://paperpile.com/c/hIfrS0/28WUL+ZR1Fh). For these requirements to be practically enforced, practical safeguards should include: (i) rigorous data governance via Data Protection Impact Assessments (DPIA), retention limits, security controls, and vendor governance [[32,34]](https://paperpile.com/c/hIfrS0/o1eGE+TEcJK); (ii) purpose limitation and technical separation between wellness data and underwriting or claims functions (unless explicit, granular opt-in is provided); (iii) opt-out mechanisms “without detriment”, preserving access to core insurance cover and standard claims handling, with pricing differences at most limited to removal of discretionary rewards rather than punitive surcharges; and (iv) regular accessibility and bias audits, plus clear consumer-facing explanations of what data are collected, what is inferred, and how to challenge decisions [[36,37]](https://paperpile.com/c/hIfrS0/28WUL+ZR1Fh). Ultimately, the integrity of an insurance contract relies on the ability to establish strict standards based on reliable evidence; wellness engagement data generally lacks the necessary context and robustness to serve as a default basis for adjudicating claims or for restricting coverage (or at least not in the absence of an explicit, proportional agreement). Similarly, in relation to employer-sponsored coverage, there must be controls ensuring that data access is provided only at an aggregate level to preserve privacy and mitigate the risk of discrimination against the respective employees.

##

## Box 5 – Evidence from Systematic Reviews and Meta-Analyses: approach and brief summaries

Searches were conducted in PubMed, Scopus, and Google Scholar using combinations of keywords including gamification, gamified intervention, game elements, health behavior, behavior change, systematic review, and meta-analysis. Reviews were considered eligible if they: (i) were explicitly described as a systematic review or meta-analysis; (ii) evaluated interventions incorporating gamification or clearly defined game elements applied in non-game contexts; and (iii) reported behavioral outcomes related to physical activity (PA). Reviews focused primarily on other health behaviors, serious games, educational gamification without behavioral outcomes, or non-health contexts were excluded.

One of the earliest review studies in the literature is a systematic review and meta-analysis of 16 randomized control trials (RCTs) involving 2,407 participants, which found that gamified interventions had a small-to-medium positive effect on PA, including moderate-to-vigorous PA (MVPA), daily step count, and active minutes (Hedges' g = 0.42, 95% Confidence Interval (CI) 0.14-0.69), with broad applicability across diverse populations [[38]](https://paperpile.com/c/hIfrS0/Bs2zz). The meta-analyses specifically revealed a significant effect of gamification on step outcomes (Hedges' g = 0.49, 95% CI 0.05-0.93), with participants in gamified interventions walking an average of 1,609.56 additional steps per day (95% CI 372.39-2,846.73) compared to those in the control group. While the long-term effects (average 14 weeks after intervention) were less pronounced (Hedges' g = 0.15, 95% CI 0.07-0.23), their persistence indicates that the benefits of gamification extend beyond the initial novelty.

Another systematic review and meta-analysis assessed the effectiveness of gamified health apps, focusing specifically on PA and cardiometabolic risk factors [[39]](https://paperpile.com/c/hIfrS0/yqdhC). This analysis of 36 RCTs involving over 10,000 participants, comparing health apps that included game elements with standard, non-gamified versions, found that users of gamified apps experienced modest but significant and clinically relevant improvements in PA and key cardiometabolic risk factors. On average, these users increased daily steps (489 steps/day, 95% CI 64 to 914) and reduced their body mass index (BMI) (−0.28 kg/m2, 95% CI −0.44 to −0.12), body weight (−0.70 kg, 95% CI −1.18 to −0.22]), body fat percentage (−1.92%, 95% CI −2.71 to −1.14), and waist circumference (−1.16 cm, 95% CI −1.93 to −0.39]).

Beyond pooled effect estimates, other reviews have examined how gamification is implemented across mobile health (mHealth) interventions and populations. In this regard, [[40]](https://paperpile.com/c/hIfrS0/cV9pt) found that gamification had been applied across different population groups, with greater prevalence among younger individuals and lower adoption among older adults and patients with chronic conditions. Goal-setting was the most commonly used game element (60%), followed by progress bars (52%), rewards (50%), points (44%), and feedback (42%). While the review suggested that gamified interventions could boost participation in PA, the results were mixed, and, due to heterogeneity across studies, only modest improvements were observed.

Population-specific evidence has also been studied, particularly among older adults, who may face distinct barriers to engagement with digital interventions. Chen et al. conducted a systematic review assessing the effectiveness and sustainability of mHealth-based gamified interventions among older adults (aged ≥60 years), synthesizing evidence from 8 trials (including RCTs and quasi-experimental studies) involving a total of 1,454 participants published up to May 2025 [[41]](https://paperpile.com/c/hIfrS0/vrk2z). The review reported that gamified interventions were generally associated with increases in daily step counts and time spent in MVPA, with goal setting and rewards identified as the most frequently used game elements. Improvements were observed across both controlled and single-arm studies, including effects reported during follow-up periods. Examination of individual trials highlighted relationships between intervention complexity, technological integration, and theoretical grounding, and the direction and magnitude of reported effects. For instance, advanced hybrid interventions grounded in Prospect Theory [[42]](https://paperpile.com/c/hIfrS0/mkTua) (a behavioral economics theory explaining that individuals are typically more motivated to avoid perceived losses than to achieve equivalent gains, particularly when outcomes are framed relative to current behavior) demonstrated the strongest effects, with [[43]](https://paperpile.com/c/hIfrS0/U9DWl) reporting a statistically significant increase in daily steps (p-value < .001) and [[44]](https://paperpile.com/c/hIfrS0/avsgO) reporting a significant improvement in MVPA (p-value = .03), despite a non-significant effect for daily steps (p-value = .08). Moderate effects on step count were observed in standalone interventions, including a Cohen's d of 0.65 (p-value = .03) reported by [[45]](https://paperpile.com/c/hIfrS0/owoub) and η² (eta squared) values ranging from 0.19 to 0.31 (p-value ≤ .04) across the series of studies by Santos et al [[46–48]](https://paperpile.com/c/hIfrS0/jLiWQ+NyJaK+bkes8). In contrast, interventions with intermediate complexity or limited theoretical grounding showed weaker or non-significant effects, including non-significant daily step outcomes (p-value = .25) reported by [[49]](https://paperpile.com/c/hIfrS0/mAsqa) and mixed findings in [[50]](https://paperpile.com/c/hIfrS0/7xSkT), where activity frequency increased (p-value = .04), but step count changes were not statistically significant.

Evidence has also been reported in younger populations. In a more recent study, a systematic review and meta-analysis evaluated the effectiveness of gamified interventions in promoting PA and reducing Sedentary Behavior (SB) among children and adolescents, synthesizing evidence from 16 RCTs involving 7,472 participants aged 6–18 years [[51]](https://paperpile.com/c/hIfrS0/0lKoR). The meta-analysis demonstrated that gamified interventions were associated with a small but statistically significant increase in MVPA (standardized mean difference (SMD) = 0.15, 95% CI 0.01-0.29; p-value = 0.04) and a reduction in BMI (SMD = 0.11, 95% CI 0.05-0.18; p-value < 0.001). In contrast, no statistically significant effects were observed for SB, daily step counts, or other PA intensities. Subgroup analyses indicated that factors such as game elements, intervention duration, and study setting moderated improvements in MVPA. The authors noted that methodological limitations, particularly the infeasibility of participant blinding, contributed to performance bias and may have influenced subjective outcome measures, underscoring the need for higher-quality trials to strengthen the evidence base.

In 2024, another meta-analysis investigated the effectiveness of gamification in influencing health behaviors (such as PA, smoking cessation, diet, etc), synthesizing evidence from 30 RCTs published between 2012 and 2023 [[52]](https://paperpile.com/c/hIfrS0/psv66). The qualitative review indicated that most studies reported favorable behavioral outcomes, although fewer than half reported mixed findings. The quantitative meta-analysis using a random-effects model, which included a total of 11,558 participants, identified a statistically significant pooled effect of gamification on behavior change (odds ratio = 1.27, 95% CI 1.20-1.33; Z = 9.06, p-value < 0.01), indicating higher odds of behavioral change in gamified interventions compared with controls. Notably, substantial between-study heterogeneity was observed (I² = 95%, χ² = 565.19, p-value < 0.01), reflecting marked variability in intervention designs, target populations, and outcome measures. Visual inspection of the funnel plot did not indicate evidence of publication bias.

Evidence from clinical populations, particularly individuals with cardiovascular diseases (CVDs), has also been examined in the literature. A systematic review and meta-analysis of mHealth–based gamified interventions to promote PA or reduce SB in individuals with CVDs synthesized findings from 6 RCTs published between 2010 and 2024 [[53]](https://paperpile.com/c/hIfrS0/FmwF6). Meta-analysis of 5 trials demonstrated a small but statistically significant short-term effect on PA following sensitivity analysis (Hedges g = 0.32, 95% CI 0.19-0.45; 95% prediction interval 0.02 to 0.62). A further meta-analysis of 3 studies indicated a small maintenance effect at follow-up (mean 2.5 months post-intervention; Hedges g = 0.20, 95% CI 0.12-0.29; 95% prediction interval −0.01 to 0.41). In absolute terms, participants in gamified interventions achieved an average of 696.96 additional steps per day compared with controls (95% CI 327.80-1066.12; 95% prediction interval −121.39 to 1515.31). Meta-regression identified feedback as the most influential game design element, followed by avatars, although the authors noted that the small number of included trials limits the certainty of these findings.

####

####

####

#### Table 1 – Definitions of the behavior change intervention functions defined in the BCW [[54]](https://paperpile.com/c/hIfrS0/4ry4W)

| **Interventions** | **Definition** | **Examples** |
| --- | --- | --- |
| Education | Increasing knowledge or understanding | Providing information to promote healthy eating |
| Persuasion | Using communication to induce positive or negative feelings or stimulate action | Using imagery to motivate increases in PA |
| Incentivization | Creating an expectation of reward | Using prize draws to induce attempts to stop smoking |
| Coercion | Creating an expectation of punishment or cost | Raising the financial cost to reduce excessive alcohol consumption |
| Training | Imparting skills | Advanced driver training to increase safe driving |
| Restriction | Using rules to reduce the opportunity to engage in the target behavior (or to increase the target behavior by reducing the opportunity to engage in competing behaviors) | Prohibiting sales of solvents to people under 18 reduce misuse for intoxication |
| Environmental restructuring | Changing the physical or social context | Providing on-screen prompts for GPs to ask about smoking behavior |
| Modeling | Providing an example for people to aspire to or imitate | Using TV drama scenes involving safe-sex practices to increase condom use |
| Enablement | Increasing means/reducing barriers to increase capability or opportunity | Behavioral support for smoking cessation, medication for cognitive deficits, surgery to reduce obesity, prostheses to promote PA |

####

#### **Table 2 – Summary of the 16 Behavior Change Technique (BCT) clusters as defined in BCT Taxonomy v1** [[55]](https://paperpile.com/c/hIfrS0/loCuW)

| **BCT cluster** | **Focus of the techniques within the cluster** | **Example** |
| --- | --- | --- |
| Goals and planning | Involves setting goals, planning actions, and reviewing behavioral targets. | Establishing a plan to walk 3000 steps in half an hour every day. |
| Feedback and monitoring | Focuses on observing behavior and providing feedback to promote change. | Using a fitness tracker or a mobile app to monitor daily steps and reviewing progress weekly. |
| Social support | Encourages the use of social networks for emotional or practical support. | Joining a support group to share experiences and strategies for quitting smoking. |
| Shaping knowledge | Provides information or instructions to increase understanding and skill. | Attending a workshop on healthy cooking techniques to improve diet. |
| Natural consequences | Highlights the health, emotional, or social outcomes of behaviors. | Reading about the long-term health risks associated with excessive alcohol consumption. |
| Comparison of behavior | Encourages comparison of one's behavior with others to motivate change. | Observing peers who have successfully adopted regular exercise routines. |
| Associations | Links behaviors with cues or contexts to build or break habits. | Sending reminders to encourage daily activity. |
| Repetition and substitution | Uses practice or replacement behaviors to encourage habit formation. | Suggesting a short walk or stretches instead of sedentary television viewing. |
| Comparison of outcomes | Involves comparing potential results of different behavioral choices. | Weighing the benefits of a balanced diet versus the risks of continued unhealthy eating. |
| Reward and threat | Applies incentives or consequences to reinforce or discourage behaviors. | Setting up a reward system for completing health-related activities. |
| Regulation | Includes strategies that modify emotional or cognitive processes to support change. | Practicing mindfulness meditation to manage stress-related eating. |
| Antecedents | Modifies the environment to make desired behaviors easier or unwanted ones harder. | Removing sugary snacks from the home to reduce temptation. |
| Identity | Targets self-concept and personal values to encourage behavior change. | Adopting the identity of an 'ex-smoker' to reinforce quitting smoking. |
| Scheduled consequences | Schedules consequences to shape behavior over time. | Arranging rewards for consistent PA streaks. |
| Self-belief | Enhances confidence in one's ability to perform the desired behavior. | Reflecting on past successes to build confidence in maintaining a new diet. |
| Covert learning | Uses imagination or observation to mentally rehearse or reflect on consequences. | Visualizing successfully navigating a social event without consuming alcohol. |

**References**

1. [Vitality. Rewards for keeping healthy. Fancy it? [cited 17 Jun 2025]. Available:](http://paperpile.com/b/hIfrS0/azS2A) <https://www.vitality.co.uk/rewards/>

2. [Vitality. Vitality to Transform Client Engagement with Evolution of Vitality Programme with launch of Pick and Play. 12 Feb 2025 [cited 17 Jun 2025]. Available:](http://paperpile.com/b/hIfrS0/1vvI) <https://www.vitality.co.uk/media/evolution-of-vitality-programme-with-launch-of-pick-and-play/>

3. [YuLife. From Steps to Science: How Gamification is Transforming Health and Life Insurance. 16 Apr 2025 [cited 17 Jun 2025]. Available:](http://paperpile.com/b/hIfrS0/2P2xl) <https://yulife.com/blog/how-gamification-transforms-group-health-life-insurance/>

4. [YuLife. YuLife Partners with MetLife UK to Launch Pre-Early Intervention Group Income Protection Solution. 7 Nov 2024 [cited 17 Jun 2025]. Available:](http://paperpile.com/b/hIfrS0/T2N0e) <https://yulife.com/news-and-events/metlife-yulife-launch-group-income-protection/>

5. [YuLife. YuLife launches Group Health Insurance provided by Bupa: here’s how it works. 22 Sep 2023 [cited 17 Jun 2025]. Available:](http://paperpile.com/b/hIfrS0/Mepfm) <https://yulife.com/blog/how-yulife-business-health-insurance-works/>

6. [YuLife. Does Gamification Still Work, or is it a Trend From the Past? 13 Feb 2025 [cited 17 Jun 2025]. Available:](http://paperpile.com/b/hIfrS0/8jtcZ) <https://yulife.com/blog/does-gamification-still-work/>

7. [Aviva. Aviva Wellbeing: Empowering employees to reach wellbeing goals. [cited 17 Jun 2025]. Available:](http://paperpile.com/b/hIfrS0/JtAoB) <https://www.aviva.co.uk/business/workplace-wellbeing/wellbeing/aviva-wellbeing/>

8. [AXA Health. Find out everything your AXA Health membership offers you. [cited 17 Jun 2025]. Available:](http://paperpile.com/b/hIfrS0/NnT7M) <https://www.axahealth.co.uk/members/member-benefits-explained/>

9. [Alyafei A, Easton-Carr R. The Health Belief Model of Behavior Change. StatPearls. Treasure Island (FL): StatPearls Publishing; 2024.](http://paperpile.com/b/hIfrS0/Vd83s)

10. [Rosenstock IM, Strecher VJ, Becker MH. Social learning theory and the Health Belief Model. Health Educ Q. 1988;15: 175–183.](http://paperpile.com/b/hIfrS0/bIIxh)

11. [Holloway A, Watson HE. Role of self-efficacy and behaviour change. Int J Nurs Pract. 2002;8: 106–115.](http://paperpile.com/b/hIfrS0/wHYU8)

12. [Schwarzer R, Warner LM. Perceived self-efficacy and its relationship to resilience. In: Prince-Embury S, Saklofske DH, editors. Resilience in children, adolescents, and adults: Translating research into practice. Springer Science + Business Media; 2013. pp. 139–150.](http://paperpile.com/b/hIfrS0/sJaIP)

13. [French DP, Olander EK, Chisholm A, Mc Sharry J. Which behaviour change techniques are most effective at increasing older adults’ self-efficacy and physical activity behaviour? A systematic review. Ann Behav Med. 2014;48: 225–234.](http://paperpile.com/b/hIfrS0/qge7P)

14. [Prestwich A, Kellar I, Parker R, MacRae S, Learmonth M, Sykes B, et al. How can self-efficacy be increased? Meta-analysis of dietary interventions. Health Psychol Rev. 2014;8: 270–285.](http://paperpile.com/b/hIfrS0/uwg7p)

15. [Olander EK, Fletcher H, Williams S, Atkinson L, Turner A, French DP. What are the most effective techniques in changing obese individuals’ physical activity self-efficacy and behaviour: a systematic review and meta-analysis. Int J Behav Nutr Phys Act. 2013;10: 29.](http://paperpile.com/b/hIfrS0/6JmBf)

16. [Newby K, Teah G, Cooke R, Li X, Brown K, Salisbury-Finch B, et al. Do automated digital health behaviour change interventions have a positive effect on self-efficacy? A systematic review and meta-analysis. Health Psychol Rev. 2021;15: 140–158.](http://paperpile.com/b/hIfrS0/iMFrN)

17. [Ashford S, Edmunds J, French DP. What is the best way to change self-efficacy to promote lifestyle and recreational physical activity? A systematic review with meta-analysis. Br J Health Psychol. 2010;15: 265–288.](http://paperpile.com/b/hIfrS0/YpEyb)

18. [Ryan RM, Deci EL. Self-determination theory and the facilitation of intrinsic motivation, social development, and well-being. Am Psychol. 2000;55: 68–78.](http://paperpile.com/b/hIfrS0/X3bF0)

19. [Ng JYY, Ntoumanis N, Thøgersen-Ntoumani C, Deci EL, Ryan RM, Duda JL, et al. Self-determination theory applied to health contexts: A meta-analysis: A meta-analysis. Perspect Psychol Sci. 2012;7: 325–340.](http://paperpile.com/b/hIfrS0/EmTrn)

20. [Fogg BJ. A behavior model for persuasive design. Proceedings of the 4th International Conference on Persuasive Technology. New York, NY, USA: ACM; 2009. doi:](http://paperpile.com/b/hIfrS0/3Jyqy)[10.1145/1541948.1541999](http://dx.doi.org/10.1145/1541948.1541999)

21. [Ryan RM, Deci EL. Intrinsic and Extrinsic Motivations: Classic Definitions and New Directions. Contemp Educ Psychol. 2000;25: 54–67.](http://paperpile.com/b/hIfrS0/VnpyQ)

22. [Ryan RM. Psychological needs and the facilitation of integrative processes. J Pers. 1995;63: 397–427.](http://paperpile.com/b/hIfrS0/T3vJI)

23. [Deci EL. Intrinsic Motivation. Springer Science & Business Media; 2012.](http://paperpile.com/b/hIfrS0/t8YlN)

24. [Seligman MEP. Helplessness: On Depression, Development, and Death. W. H. Freeman; 1992.](http://paperpile.com/b/hIfrS0/md1J7)

25. [Ntoumanis N, Ng JYY, Prestwich A, Quested E, Hancox JE, Thøgersen-Ntoumani C, et al. A meta-analysis of self-determination theory-informed intervention studies in the health domain: effects on motivation, health behavior, physical, and psychological health. Health Psychol Rev. 2021;15: 214–244.](http://paperpile.com/b/hIfrS0/roA83)

26. [Oudeyer P-Y, Kaplan F. What is Intrinsic Motivation? A Typology of Computational Approaches. Front Neurorobot. 2007;1: 6.](http://paperpile.com/b/hIfrS0/rhxuJ)

27. [Kim TW, Werbach K. More than just a game: ethical issues in gamification. Ethics and Information Technology. 2016;18: 157–173.](http://paperpile.com/b/hIfrS0/rmADG)

28. [Yang H, Li D. Understanding the dark side of gamification health management: A stress perspective. Inf Process Manag. 2021;58: Article 102649.](http://paperpile.com/b/hIfrS0/5HCxP)

29. [Durieux BN, DeCamp M, Lindvall C. 21st Century Cures Act: ethical recommendations for new patient-facing products. J Am Med Inform Assoc. 2022;29: 1818–1822.](http://paperpile.com/b/hIfrS0/JltIZ)

30. [Arora C, Razavian M. Ethics of Gamification in Health and Fitness-Tracking. Int J Environ Res Public Health. 2021;18. doi:](http://paperpile.com/b/hIfrS0/cyq12)[10.3390/ijerph182111052](http://dx.doi.org/10.3390/ijerph182111052)

31. [Equality Act 2010. In: King’s Printer of Acts of Parliament [Internet]. [cited 17 Dec 2025]. Available:](http://paperpile.com/b/hIfrS0/xUQN0) <https://www.legislation.gov.uk/ukpga/2010/15/notes/contents>

32. [What are the rules on special category data? In: ICO [Internet]. 9 Oct 2025 [cited 17 Dec 2025]. Available:](http://paperpile.com/b/hIfrS0/o1eGE) <https://ico.org.uk/for-organisations/uk-gdpr-guidance-and-resources/lawful-basis/special-category-data/what-are-the-rules-on-special-category-data/>

33. [What are the conditions for processing? In: ICO [Internet]. 22 Sep 2025 [cited 17 Dec 2025]. Available:](http://paperpile.com/b/hIfrS0/N0ojm) <https://ico.org.uk/for-organisations/uk-gdpr-guidance-and-resources/lawful-basis/special-category-data/what-are-the-conditions-for-processing/>

34. [A guide to the data protection principles. In: ICO [Internet]. 4 Dec 2024 [cited 17 Dec 2025]. Available:](http://paperpile.com/b/hIfrS0/TEcJK) <https://ico.org.uk/for-organisations/uk-gdpr-guidance-and-resources/data-protection-principles/a-guide-to-the-data-protection-principles/>

35. [PS22/9: A new Consumer Duty. In: FCA [Internet]. 2 Dec 2021 [cited 17 Dec 2025]. Available:](http://paperpile.com/b/hIfrS0/n6uGZ) <https://www.fca.org.uk/publications/policy-statements/ps22-9-new-consumer-duty>

36. [Automated decision-making and profiling. In: ICO [Internet]. 12 Aug 2025 [cited 17 Dec 2025]. Available:](http://paperpile.com/b/hIfrS0/28WUL) <https://ico.org.uk/for-organisations/uk-gdpr-guidance-and-resources/individual-rights/automated-decision-making-and-profiling/>

37. [Explaining decisions made with AI. In: ICO [Internet]. 15 Jul 2025 [cited 17 Dec 2025]. Available:](http://paperpile.com/b/hIfrS0/ZR1Fh) <https://ico.org.uk/for-organisations/uk-gdpr-guidance-and-resources/artificial-intelligence/explaining-decisions-made-with-artificial-intelligence/>

38. [Mazeas A, Duclos M, Pereira B, Chalabaev A. Evaluating the Effectiveness of Gamification on Physical Activity: Systematic Review and Meta-analysis of Randomized Controlled Trials. J Med Internet Res. 2022;24: e26779.](http://paperpile.com/b/hIfrS0/Bs2zz)

39. [Nishi SK, Kavanagh ME, Ramboanga K, Ayoub-Charette S, Modol S, Dias GM, et al. Effect of digital health applications with or without gamification on physical activity and cardiometabolic risk factors: a systematic review and meta-analysis of randomized controlled trials. EClinicalMedicine. 2024;76: 102798.](http://paperpile.com/b/hIfrS0/yqdhC)

40. [Xu L, Shi H, Shen M, Ni Y, Zhang X, Pang Y, et al. The Effects of mHealth-Based Gamification Interventions on Participation in Physical Activity: Systematic Review. JMIR Mhealth Uhealth. 2022;10: e27794.](http://paperpile.com/b/hIfrS0/cV9pt)

41. [Chen L, Jang F, Li M, Zong W, Yu H. Effectiveness of mHealth-based gamified interventions on physical activity in older adults: Systematic review. JMIR Aging. 2025;8: e78686.](http://paperpile.com/b/hIfrS0/vrk2z)

42. [Kahneman D, Tversky A. Prospect theory: An analysis of decision under risk. Handbook of the Fundamentals of Financial Decision Making. WORLD SCIENTIFIC; 2013. pp. 99–127.](http://paperpile.com/b/hIfrS0/mkTua)

43. [Greysen SR, Oon AL, Harkins K, Rareshide C, Mondal A, Patel MS, et al. Effect of gamification with a support partner to increase physical activity in older adults at risk for Alzheimer’s disease: The STEP 4Life randomized clinical trial. Alzheimers Dement. 2024;20: 5450–5459.](http://paperpile.com/b/hIfrS0/U9DWl)

44. [Fanaroff AC, Patel MS, Chokshi N, Coratti S, Farraday D, Norton L, et al. Effect of Gamification, Financial Incentives, or Both to Increase Physical Activity Among Patients at High Risk of Cardiovascular Events: The BE ACTIVE Randomized Controlled Trial. Circulation. 2024;149: 1639–1649.](http://paperpile.com/b/hIfrS0/avsgO)

45. [Martinho D, Crista V, Carneiro J, Matsui K, Corchado JM, Marreiros G. Effects of a gamified agent-based system for personalized elderly care: Pilot usability study. JMIR Serious Games. 2023;11: e48063.](http://paperpile.com/b/hIfrS0/owoub)

46. [Santos LH, Okamoto K, Hiragi S, Yamamoto G, Sugiyama O, Aoyama T, et al. Pervasive game design to evaluate social interaction effects on levels of physical activity among older adults. J Rehabil Assist Technol Eng. 2019;6: 2055668319844443.](http://paperpile.com/b/hIfrS0/jLiWQ)

47. [Santos LHDO, Okamoto K, Funghetto SS, Cavalli AS, Hiragi S, Yamamoto G, et al. Effects of social interaction mechanics in pervasive games on the physical activity levels of older adults: Quasi-experimental study. JMIR Serious Games. 2019;7: e13962.](http://paperpile.com/b/hIfrS0/NyJaK)

48. [Santos LHDO, Okamoto K, Otsuki R, Hiragi S, Yamamoto G, Sugiyama O, et al. Promoting physical activity in Japanese older adults using a social pervasive game: Randomized controlled trial. JMIR Serious Games. 2021;9: e16458.](http://paperpile.com/b/hIfrS0/bkes8)

49. [Randriambelonoro M, Perrin Franck C, Herrmann F, Carmona GA, Geissbuhler A, Graf C, et al. Gamified physical rehabilitation for older adults with musculoskeletal issues: Pilot noninferiority randomized clinical trial. JMIR Rehabil Assist Technol. 2023;10: e39543.](http://paperpile.com/b/hIfrS0/mAsqa)

50. [Kawaguchi K, Nakagomi A, Ide K, Kondo K. Effects of a mobile app to promote Social Participation on older adults: Randomized controlled trial. J Med Internet Res. 2024;26: e64196.](http://paperpile.com/b/hIfrS0/7xSkT)

51. [Wang M, Xu J, Zhou X, Li X, Zheng Y. Effectiveness of gamification interventions to improve physical activity and sedentary behavior in children and adolescents: Systematic review and meta-analysis. JMIR Serious Games. 2025;13: e68151.](http://paperpile.com/b/hIfrS0/0lKoR)

52. [Alzghoul B. The effectiveness of gamification in changing health-related behaviors: A systematic review and meta-analysis. Open Public Health J. 2024;17. doi:](http://paperpile.com/b/hIfrS0/psv66)[10.2174/0118749445234806240206094335](http://dx.doi.org/10.2174/0118749445234806240206094335)

53. [Yu T, Parry M, Yu T, Xu L, Wu Y, Zeng T, et al. Effectiveness of mobile health-based gamification interventions for improving physical activity in individuals with cardiovascular diseases: Systematic review and meta-analysis of randomized controlled trials. JMIR Serious Games. 2025;13: e64410.](http://paperpile.com/b/hIfrS0/FmwF6)

54. [Michie S, van Stralen MM, West R. The behaviour change wheel: a new method for characterising and designing behaviour change interventions. Implement Sci. 2011;6: 42.](http://paperpile.com/b/hIfrS0/4ry4W)

55. [Michie S, Richardson M, Johnston M, Abraham C, Francis J, Hardeman W, et al. The behavior change technique taxonomy (v1) of 93 hierarchically clustered techniques: building an international consensus for the reporting of behavior change interventions. Ann Behav Med. 2013;46: 81–95.](http://paperpile.com/b/hIfrS0/loCuW)
